# Serologic Survey of Pandemic (H1N1) 2009 Virus, Guangxi Province, China

**DOI:** 10.3201/eid1511.090868

**Published:** 2009-11

**Authors:** Honglin Chen, Yong Wang, Wei Liu, Jinxia Zhang, Baiqing Dong, Xiaohui Fan, Menno D. de Jong, Jeremy Farrar, Steven Riley, Gavin J. D. Smith, Yi Guan

**Affiliations:** Shantou University Medical College, Shantou, People’s Republic of China (H. Chen, J. Zhang, S. Riley, G.J.D. Smith, Y. Guan); The University of Hong Kong, Hong Kong Special Administrative Region, People’s Republic of China (H. Chen, J. Zhang, S. Riley, G.J.D. Smith, Y Guan); Guangxi Medical University, Nanning, People’s Republic of China (Y. Wang, X. Fan); Guangxi Center for Disease Control and Prevention, Nanning (W. Liu, B. Dong); Hospital for Tropical Diseases, Ho Chi Minh City, Vietnam (M.D. de Jong, J. Farrar); 1 These authors contributed equally to this article.

**Keywords:** Influenza, influenza virus, immunity, vaccination, community outbreak, epidemiology, China, expedited, letter

**To the Editor:** Since mid-April 2009, a new influenza A virus (H1N1), now called pandemic (H1N1) 2009 virus, has caused influenza outbreaks in humans in North America (*1*) and a worldwide pandemic (*2**–**4*). Human pandemics occur when a new virus subtype emerges that is capable of human-to-human transmission in a population with little or no neutralizing antibodies to the new virus (*4*).

The current outbreak presents the first opportunity to directly observe this process. We used hemagglutination inhibition (HI) and virus neutralization (VN) assays to detect antibodies in 4,043 serum samples from residents (7–84 years of age) of 2 counties in Guangxi Province, People’s Republic of China, collected during July–August 2008. These persons were mostly farmers who lived in rural areas. Serum samples were obtained, transported, and frozen at –80°C as described (*5*). No participants had a history of vaccination against seasonal influenza. Antibodies were also detected in another 22 persons (<40 years of age) in Shantou, Guangdong Province, who had received 3 vaccinations for seasonal influenza since 2006.

Influenza viruses used in this study were A/California/04/2009 (H1N1; CA04), A/Brisbane/59/2007 (H1N1; B59), and A/swine/Hong Kong/915/2004 (H1N2; Sw915). CA04 and B59 were kindly provided by the World Health Organization Collaborating Centers for Reference and Research on Influenza (Atlanta, GA, USA, and Parkville, Victoria, Australia). Sw915 was isolated from pigs by our laboratory. Seven of 8 genomic segments of Sw915 were located in a sister lineage to the current outbreak; this strain is the most closely related swine virus to CA04 identified to date (*6*). All serum samples were treated with a receptor-destroying enzyme and absorbed with fresh turkey erythrocytes to remove nonspecific inhibitors before the assays. All samples were tested by HI and VN assays according to standard protocols (*5*).

Screening by HI assay showed that 70 samples were positive (titers >40) for CA04 (Table). Examination by VN assay showed that of 70 HI-positive serum samples, 12 had detectable neutralizing antibodies to CA04 (positive rate 0.3%). Of these VN-positive samples, 10 had titers of 40–80 and only 2 had neutralizing antibody titers >160 (Table). The 12 persons from whom the samples were obtained were 30–60 years of age. In contrast with findings from a recent serologic survey of a US population (*7*), our results showed that none of the 583 persons >60 years of age in our study was VN seropositive for CA04.

All 70 HI-positive samples for CA04 were also screened for neutralizing antibodies against Sw915. Thirteen samples collected from persons 40–84 years of age were VN positive (titers 40–160). Of these 13 samples, 5 were positive (VN titer >40) for CA04 and 8 were negative. However, 7 CA04 VN-positive samples were negative for Sw915. These findings suggest that some cross-reactivity exists between CA04 and other Sw915-like H1 subtype viruses circulating in the pig population in southern China, and that sporadic human infection with H1 swine viruses has occurred in rural China, where exposure to pigs is common.

In contrast, screening all 4,043 serum samples with A/Brisbane/59/2007 showed that 159 (3.9%) samples had HI titers >40, of which 116 (2.9%) had neutralizing antibodies (titer >40) (Table). Only 3 serum samples from persons >60 years of age were VN positive for B59. Because the study group was not vaccinated, these results likely reflect natural infection rates for seasonal influenza virus (H1N1). The 22 serum samples from vaccinated persons had no neutralizing antibodies against CA04, but all had high seroconversion rates for B59 (Table).

Our results suggest that most persons in our study population from Guangxi, China, are seronegative for pandemic (H1N1) 2009 virus (*1*). Serum samples from only 0.3% of persons tested neutralized the novel CA/04 strain. This finding contrasts with findings from the United States that serum samples from ≈11% of unvaccinated persons had antibodies against CA04 (*7*). Furthermore, all CA04-positive persons in our study were <60 years of age; the US study reported a 33% seropositive rate for this age group.

These differences may have been caused by the high proportion of seasonal influenza vaccination coverage in the United States when compared with results form our unvaccinated population from southern China. Therefore, we suggest that vaccination against seasonal influenza, rather than exposure to older, seasonal, influenza viruses (H1N1), which may be genetically and antigentically similar to pandemic (H1N1) 2009 virus, as suggested (*7*), might have generated partial protection against this new virus. No persons in our vaccinated control group had neutralizing antibodies against CA04.

We hypothesize that the absence of neutralizing antibodies in our control group, all of whom had been vaccinated 3 times, suggests that prolonged and repeated vaccination is required for partial immunity to CA04 or that older vaccines may confer some degree of protection. If these serologic differences are indicative of increased susceptibility, we would expect higher infection attack rates in largely unvaccinated populations than in vaccinated populations in countries such as China.

**Table:** Serum antibodies to pandemic (H1N1) 2009 virus A/California/04/2009 and influenza A virus (H1N1) A/Brisbane/59/2007 in unvaccinated and vaccinated persons, Guangxi Province, People’s Republic of China*

Virus, titer	No. (%) unvaccinated persons, n = 4,043			No. (%) vaccinated† persons, n = 22	
	HI	VN		HI	VN
Pandemic (H1N1) 2009 virus A/California/04/2009					
40–80	64	10		0	0
>160	6	2		0	0
Total	70 (1.7)	12 (0.3)		0	0
Influenza A virus (H1N1) A/Brisbane/59/2007					
40–80	131	64		9	9
>160	28	52		10	11
Total	159 (3.9)	116 (2.9)		19 (86)	20 (91)

*HI, hemagglutination inhibition; VN, virus neutralization. †Persons were vaccinated starting in 2006 with influenza virus (H1N1) strains A/New Caledonia/20/1999, A/Solomon Islands/3/2006, and A/Brisbane/59/2007.
